# Cloning of a Recombinant Plasmid Encoding PpSP42 Protein Fragment of *Phlebotomus papatasi* and expressing it in HEK-293T Eukaryotic Cell

**Published:** 2019-07

**Authors:** Touraj MIANDOABI, Fariborz BAHRAMI, Vahideh MOEIN VAZIRI, Soheila AJDARY

**Affiliations:** 1. Department of Parasitology and Mycology, Shahid Beheshti University of Medical Sciences, Tehran, Iran; 2. Department of Immunology, Pasteur Institute of Iran, Tehran, Iran

## Dear Editor-in-Chief

*Leishmania* parasites cause a variety of infectious diseases from cutaneous to visceral, in many developing countries around the world (1). *Leishmania* species are transmitted to their vertebrate hosts by infected sand fly bites. They have acquired means to manipulate the immune system of their hosts during their long co-evolutionary existence. Moreover, compounds attributed to the sand fly vector have been recognized to have active roles throughout the infection (2, 3). Among them are biomolecules found in the vectors’ saliva composed of anticlotting, antiplatelet, and vasodilatory proteins and reagents. Such compounds are injected into the blood-feeding sites during transmission as well as during noninfectious feedings (2, 4). The existing proteins in saliva of the sand fly vectors are shown to shift the adaptive immune response from a Th1 to a Th2 cell-mediated immune response by increasing the production of IL-4 and IL-6 cytokines as well as by inhibiting the secretion of TNFα, IL-γ, IL-12 cytokines and nitric oxide by the effector cells (5).

Here, we used a partial segment (79% of an intron-less coding gene) of salivary gland protein 42 (PpSPP42) of *Phlebotomus papatasi*, the sand fly vector of *L. major*, one of the causative agents of cutaneous leishmaniasis (CL) in Iran. Although no known function has been reported for PpSPP42 since its identification in 2001 (4), its homolog, namely LJM11 salivary gland protein of *Lutzomyia longipalpis* (the vector of *L. brasiliensis* in the New World), has been shown to confer long-term protection against CL (6). Genomic DNA of *Ph. papatasi* sand fly trapped in Kaleibar region of East Azerbaijan (Iran) was used as a template along with the following forward and reverse primers, designed based on DNA sequences confirmed at Pasteur Institute of Iran (GenBank by accession number: KX611849.1). The forward primer (5′- ATCAGAATTCCACCATGGCGGCTTACGATTCAGGAAATATTG-3′) contained an *EcoR*I (GAATTC) restriction site and a Kozak translation initiation sequence consensus (CACCATGGCG) and the reverse primer (5′ATCACTCGAGTCACATAATGTCTGTGCCAAAATTGAAG-3′) had an *Xho*I (CTCGAG) restriction site and a stop codon (TCA). These primers were used to amplify a 955- bp amplicon.

**Fig. 1:**
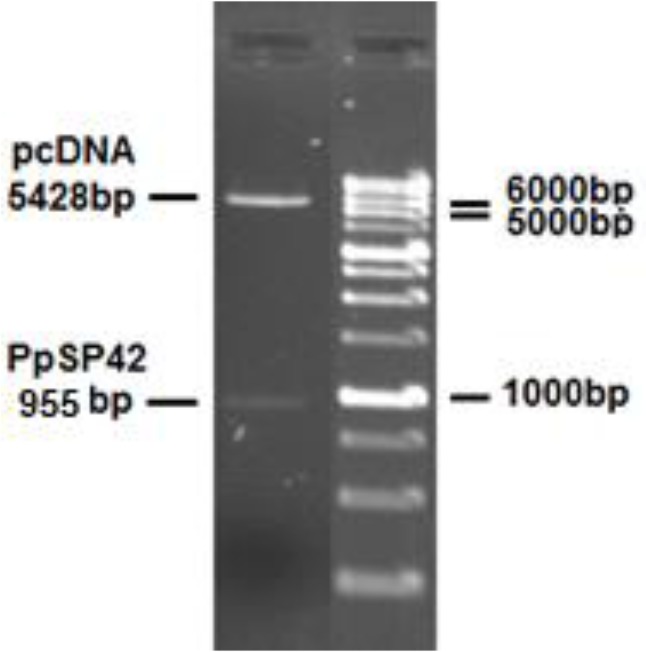
pcPpSP42 double-digested with *EcoR*I and *Xho*I (expected digested fragments: 955 bp for PpSP42 insert and 5428 bp for pcDNA3+ vector)

The PpSP42 amplicon was TA-cloned (InsTAclone™ PCR product cloning kit, Thermo Scientific, USA) and then was subcloned into pCDNA3.1+ (Invitrogen) eukaryotic expression vector, following double-digestion with *EcoR*I and *Xho*I restriction enzymes (Fermentas, Germany). The recombinant plasmid was transformed into competent *Escherichia coli* Top10 cells. The obtained pcPpSP42 plasmid was verified by restriction digestions (Fig. 1).

**Fig. 2:**
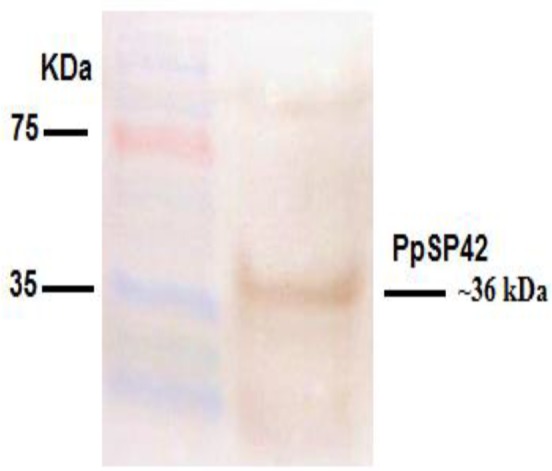
Western-blot of clear lysate of HEK-293T cell-line, transfected with pcPpSP42 construct after 72-h incubation period, detected by polyclonal murine anti-PP42 antibody

The integrity of the construct and the lack of deletions or mutations were verified by nucleotide sequencing (Gen Fanavaran Co.). Plasmid pcPpSP42 was successfully transfected into the HEK-293T cells (4×10^5^ cells/2ml medium/well) using X-tremeGENE HP DNA Transfection Reagent (Roche, Germany) and the overexpression of partial PpSP42 protein with an approximate Mw of ∼36 kDa was verified by SDS-PAGE and Western-Blotting using polyclonal anti- PpSP42 antibody, as shown in Fig. 2.

The identification of new antigens, capable of conferring long-lasting Th1 immune responses against the intracellular *Leishmania* parasites, may play the most essential role for prophylactic control of leishmaniasis. DNA vaccines are a promising option in this regard which can trigger Th1 cell signaling route by induction of CD4+ and CD8+ T-cells like an adjuvant (7).

The constructed eukaryotic expression vector in this study was capable of expressing SpPP42 in mammalian HEK cell line. The protective efficacy of this construct as a DNA vaccine against L. major-inflicted zoonotic CL remains will be evaluated using a murine model of the infection.
